# A new cave amphipod, *Pseudocrangonyx wonkimi* sp. nov. (Crustacea, Amphipoda, Pseudocrangonyctidae), from the Korean Peninsula

**DOI:** 10.3897/zookeys.960.53564

**Published:** 2020-08-17

**Authors:** Chi-Woo Lee, Ko Tomikawa, Gi-Sik Min

**Affiliations:** 1 Department of Biological Sciences, Inha University, Incheon 22212, South Korea Inha University Incheon South Korea; 2 Department of Science Education, Graduate School of Education, Hiroshima University, Higashihiroshima 739-8524, Japan Hiroshima University Higashihiroshima Japan

**Keywords:** COI, Crangonyctoidea, groundwater, morphology, South Korea

## Abstract

A new species of pseudocrangonyctid amphipod, *Pseudocrangonyx wonkimi***sp. nov.**, was found in the groundwater of a cave in the southwestern Korean Peninsula. *Pseudocrangonyx wonkimi***sp. nov.** is morphologically most closely related to *P. joolaei*Lee et al., 2020. However, *P. wonkimi* is clearly distinguished from *P. joolaei* by lacking sternal gills, fewer setae on maxilla 1 inner plate, fewer serrate robust setae on the carpus of the gnathopods, lacking bifid setae on the inner ramus of pleopod 3, and fewer articles of rami on pleopod 3. We also determined sequences of mitochondrial cytochrome c oxidase subunit I (COI) of *P. wonkimi***sp. nov.** for molecular diagnosis. From the molecular analysis based on COI sequences, *P. wonkimi* showed the closest relationship with *P. joolaei* with 15.1% genetic distance.

## Introduction

The genus *Pseudocrangonyx* Akatsuka & Komai, 1922 is one of the stygobitic groups of groundwater environments in Eastern Asia (Holsinger 1994). Species of the genus *Pseudocrangonyx* are known from subterranean waters and springs in the Korean Peninsula, Japan, Eastern China, and the Far East of Russia (Sidorov and Holsinger 2007; Tomikawa et al. 2016; Zhao and Hou 2017). So far, the genus contains 27 species (Lee et al. 2020), four of which have been recorded in the Korean Peninsula: *P. asiaticus* Uéno, 1934; *P. coreanus* Uéno, 1966; *P. daejeonensis*Lee et al., 2018; and *P. joolaei*Lee et al., 2020.

Although only four species have been recorded in Korea, it is possible that the specific diversity of the genus *Pseudocrangonyx* in the Korean Peninsula may have been underestimated. This is because there are about 1,000 natural caves in South Korea (Kim et al. 2004), many of which are known to be inhabited by unidentified species of *Pseudocrangonyx*. In addition, as mentioned in previous studies (Uéno 1966; Lee et al. 2020), *P. asiaticus*, which is distributed in various regions of Korea, includes cryptic species.

Recently, we found an unidentified species of *Pseudocrangonyx* collected from a cave in the southwestern part of the Korean Peninsula. Based on the results of the morphological examination of these specimens, we herein describe and illustrate them as representing a new species. These specimens were also confirmed to represent a distinct new species through molecular analysis using the mitochondrial cytochrome c oxidase subunit I (COI) gene.

## Methods

### Sample collection and morphological examination

**Figure 1.**
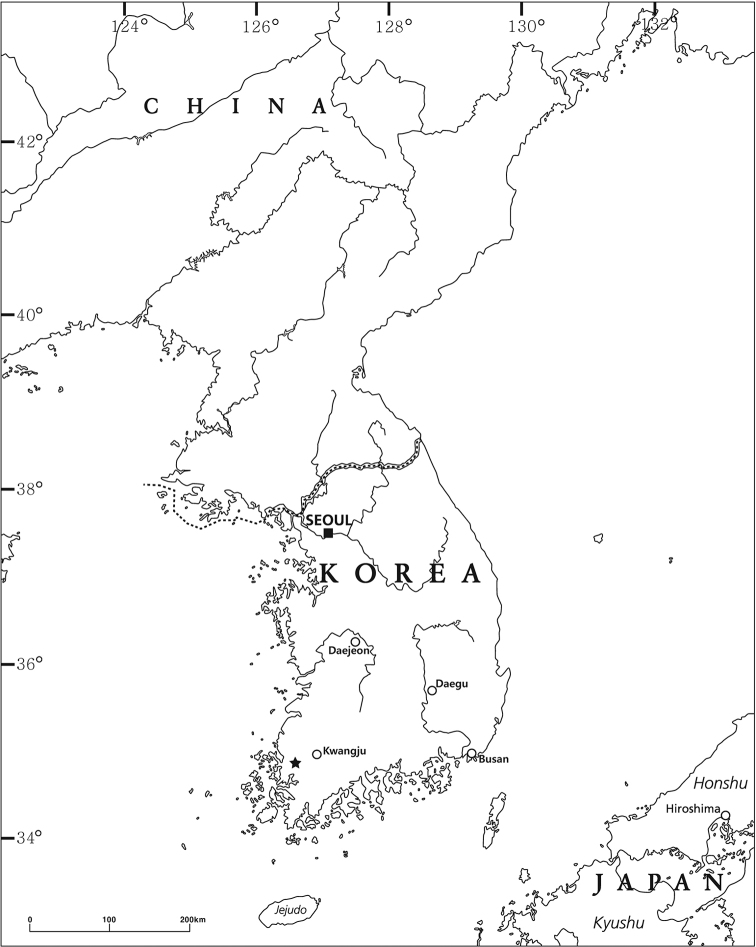
Map marking with a star the collection locality of the specimens examined in this study.

*Pseudocrangonyx* specimens were collected from the groundwater of Jungchangjin Cave, Yongseong-ri, Daedong-myeon, Hampyeong-gun, Jeollanam-do, South Korea (Fig. 1), using a fine-meshed hand net. A small pool where specimens were collected is 10 m from the entrance to the cave. Specimens were fixed and preserved in 99% ethanol. All appendages of the specimens were dissected in 80% ethanol and mounted in gum-chloral medium on glass slides under a stereomicroscope (Olympus SZX7). The specimens were examined using a light microscope (Nikon Eclipse Ni) and illustrated with the aid of a drawing tube. Body length (BL, to the nearest 0.1 mm) was measured from the tip of the rostrum to the base of the telson, along the curvature of the dorsal surface. The nomenclature of the setal patterns on the mandibular palp follows Stock (1974). The specimens examined in this study have been deposited in the collection of the Nakdonggang National Institute of Biological Resources, South Korea (NNIBR).

### Molecular analysis

Genomic DNA was extracted from the muscles of the appendages of two Korean *Pseudocrangonyx* specimens using LaboPass Tissue Mini (Cosmo GENETECH, Seoul, South Korea), according to the manufacturer’s instructions. The primer sets used for polymerase chain reaction (PCR) followed Tomikawa et al. (2016). Molecular analyses were performed using the COI sequences aligned by Geneious 8.1.9 (Biomatters, Auckland, New Zealand). Phylogenetic tree was constructed using maximum likelihood (ML) and Bayesian inference (BI). ML analysis was performed using RAxML v. 8.2.10 (Stamatakis 2014) with the substitution model set as GTRCAT, immediately after nonparametric bootstrapping conducted with 1,000 replicates. The best fit-partitioning scheme for the ML analysis was identified with the Akaike information criterion using PartitionFinder v. 2.1.1 (Lanfear et al. 2017) with the “greedy” algorithm. BI and posterior probabilities were estimated using MrBayes v. 3.2.6 (Ronquist et al. 2012). Two independent runs of four Markov chains were conducted for one million generations, and the tree was sampled every 100 generations. The parameter estimates and convergence were checked using Tracer v. 1.7.1 (Rambaut et al. 2018).

## Systematics

**Family Pseudocrangonyctidae Holsinger, 1989**

**Genus *Pseudocrangonyx* Akatsuka & Komai, 1922**

### Pseudocrangonyx wonkimi sp. nov.

Taxon classificationAnimaliaAmphipodaPseudocrangonyctidae

0B1D07DE-F8B9-5FE2-8338-9315CF239EE4

http://zoobank.org/4C4AD30E-D4BC-49A2-9E44-C4DD6ED2B33E

#### Material examined.

***Holotype***: Female (NNIBRIV35119, BL = 8.9 mm), South Korea, Jeollanam-do, Hampyeong-gun, Daedong-myeon, Yongseong-ri, Jungchangjin Cave (35°6.05'N, 126°31.99'E), 17.II.2017, Yong Gun Choi leg.

***Paratypes***: 1 male (NNIBRIV36158, BL = 8.3 mm), 1 female (NNIBRIV36157, BL = 8.9 mm), collection data same as for the holotype.

#### Diagnosis.

Female larger than male; antennal sinus with rounded angle; eyes absent; pereonites 3–5 with short dorsal setae; sternal gill absent; antenna 1 shorter than body length; antenna 2 with calceoli in both sexes; mandible palp article 3 longer than article 2; maxilla 1 inner plate with 4 plumose setae; maxilla 2 inner plate with oblique inner row of 4 setae; pleopod peduncles with anterodistal setae, inner margin of pleopods 1 and 2 inner rami with bifid setae; uropod 1 outer ramus with 2 marginal robust setae; uropod 3 terminal article of the outer ramus shorter than adjacent spines.; telson cleft for 25.0–27.4%.

#### Description.

**Figure 2.**
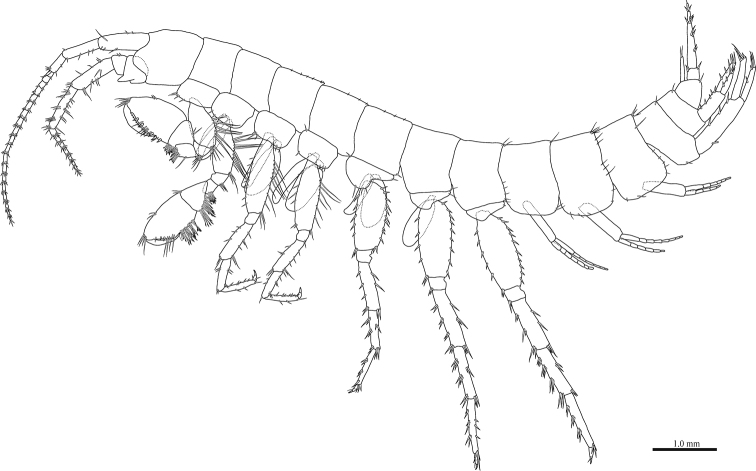
*Pseudocrangonyx wonkimi* sp. nov., holotype, female (BL = 8.9 mm). Habitus, lateral view.

**Female** (NNIBRIV35119, 8.9 mm). Head (Fig. 2) without setae; rostrum short; lateral cephalic lobe rounded; antennal sinus shallow with rounded angle; eyes absent. Pereonites 3–5 with short dorsal setae; dorsal margin of pereonite 7 with long setae. Dorsal margins of pleonites 1–3 with long setae (Fig. 2). Posterior margin and posteroventral corner of epimeral plate 1 each with seta; ventral and posterior margins of plate 2 with 3 and 4 setae, respectively, posteroventral corner with seta; ventral and posterior margins of plate 3 with 2 and 4 setae, respectively, posteroventral corner subquadrate with seta (Fig. 2). Dorsal margin of urosomites 1 and 2 with seta, urosomite 3 lacking dorsal setae. Ventral margin of urosomite 1 with seta (Fig. 2).

**Figure 3.**
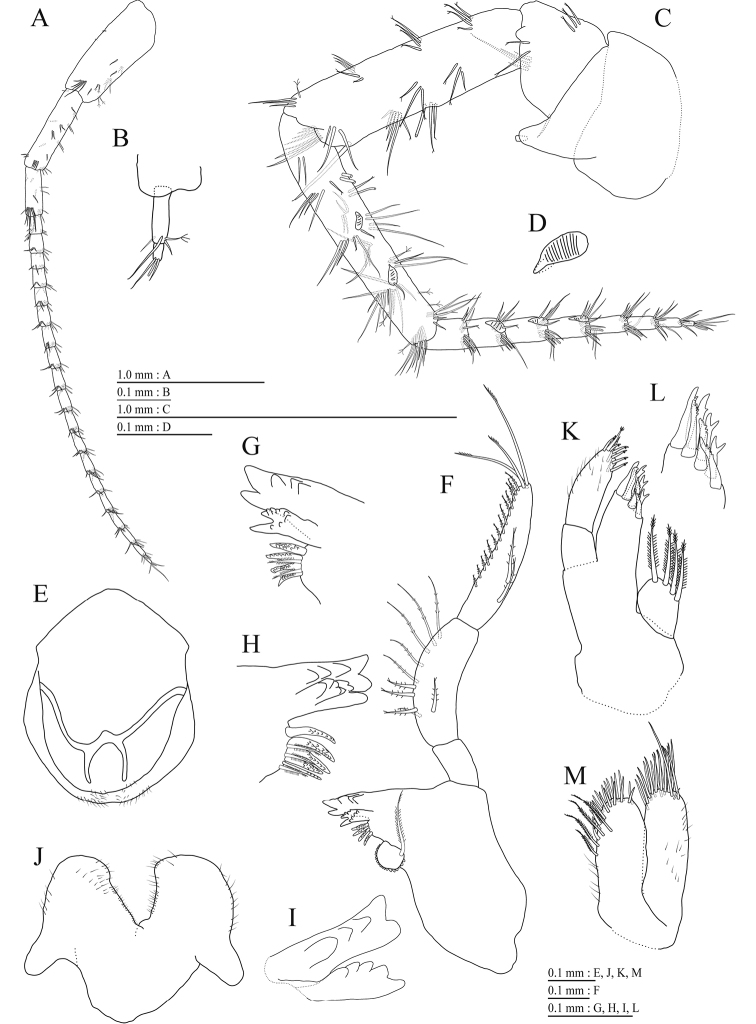
*Pseudocrangonyx wonkimi* sp. nov., holotype, female (BL = 8.9 mm): **A–H, J–M** paratype, female (BL = 8.9 mm): **I**. **A** Antenna 1, lateral view **B** accessory flagellum of antenna 1, lateral view **C** antenna 2, medial view **D** calceolus of antenna 2, medial view **E** upper lip, anterior view **F** right mandible, medial view **G** incisor and lacinia mobilis process of right mandible, medial view **H** incisor and lacinia mobilis process of left mandible, medial view **I** incisor process of left mandible, medial view **J** lower lip, dorsal view **K** maxilla 1, dorsal view **L** apical robust setae on outer plate of maxilla 1, dorsal view **M** maxilla 2, dorsal view.

Antenna 1 (Fig. 3A) 0.47 times as long as body length, peduncular articles 1–3 in length ratio of 1.0 : 0.8 : 0.4; accessory flagellum (Fig. 3B) 2-articulate, terminal article with 2 setae and aesthetascs; primary flagellum 1.5 times as long as peduncular articles 1–3 combined, 19-articulate, 1 aesthetasc on some articles. Antenna 2 (Fig. 3C, D) 0.65 times as long as antenna 1; peduncular article 5 with 2 calceoli; flagellum 0.53 times as long as peduncular articles 4 and 5 combined, consisting of 8 articles, flagellum articles 2–4 with calceolus.

**Figure 4.**
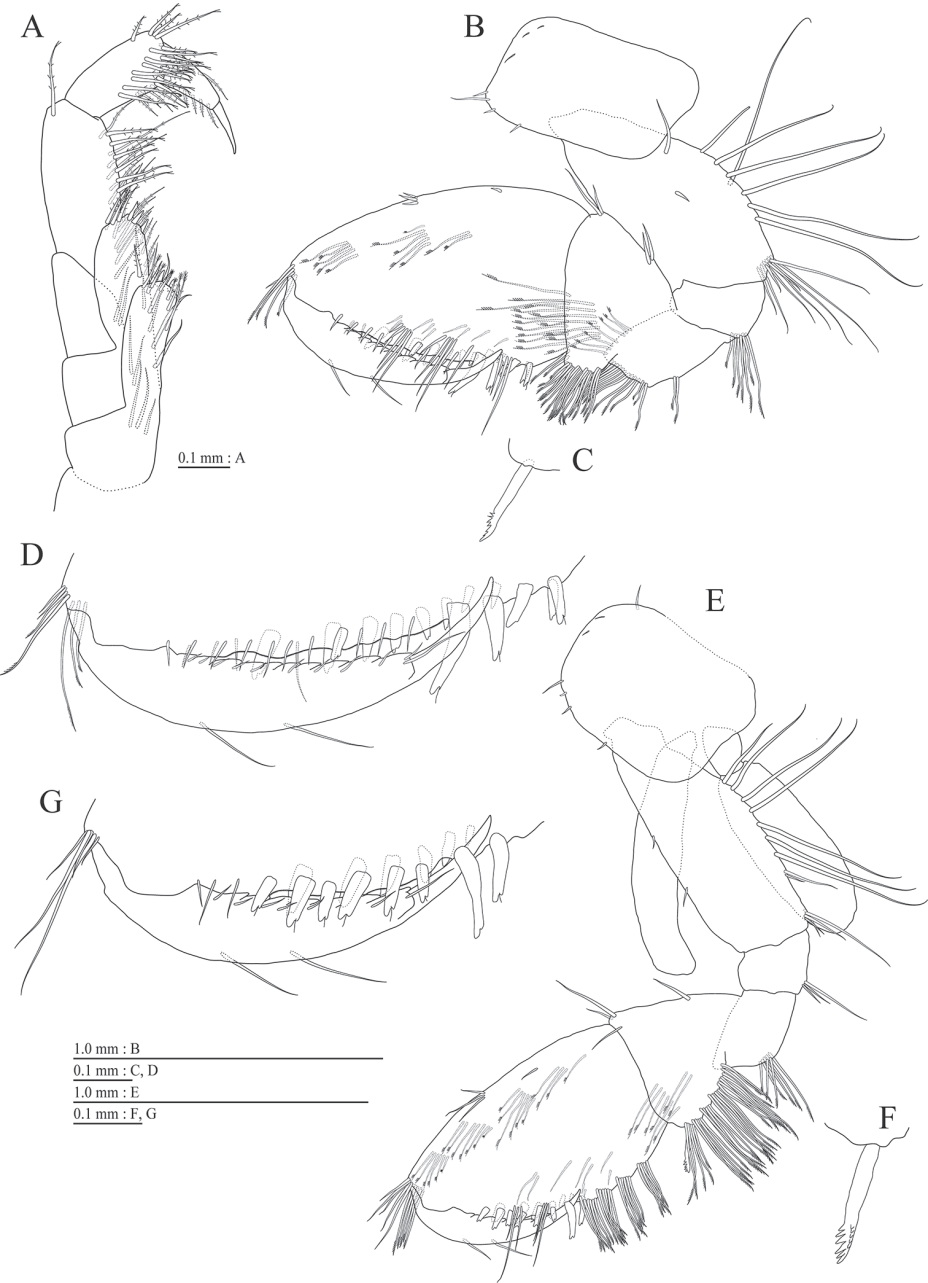
*Pseudocrangonyx wonkimi* sp. nov., holotype, female (BL = 8.9 mm). **A** Maxilliped, dorsal view **B** gnathopod 1, lateral view **C** serrate seta on posterodistal corner of carpus of gnathopod 1, lateral view **D** palmar margin of propodus and dactylus of gnathopod 1, lateral view **E** gnathopod 2, lateral view **F** serrate seta on posterodistal corner of carpus of gnathopod 2, lateral view **G** palmar margin of propodus and dactylus of gnathopod 2, lateral view.

Upper lip (Fig. 3E) with rounded anterior margin, with fine setae. Mandibles (Fig. 3F–H) with left and right incisors 5-dentate; left lacinia mobilis 5-dentate, right lacinia bifid, with many teeth; molar process triturative; accessory setal rows of left and right mandibles each with 5- and 4- pectinate setae; palp 3-articulate, article 3 with 2 A-, 14 D-, and 3 E-setae. Lower lip (Fig. 3J) with broad outer lobes with fine setae, mandibular process of outer lobe rounded apically; inner lobes indistinct. Maxilla 1 (Fig. 3K, L) with inner and outer plates, and palp; inner plate subquadrate with 4 plumose setae; outer plate subrectangular with 7 serrate teeth apically; palp 2-articulate, longer than outer plate, article 2 with weakly plumose 3 apical and 4 subapical robust setae. Maxilla 2 (Fig. 3M) with oblique inner row of 4 setae on inner plate. Maxilliped (Fig. 4A) with inner and outer plates, and palp; inner plate with 3 apical robust setae; outer plate with 6 apical plumose setae, 3 subapical robust setae, and some medial setae; palp 4-articulate, medial margin of article 2 lined with setae, article 4 with claw.

Gnathopod 1 (Fig. 4B, C) with subrectanqular coxal plate, bearing setae on anterior margin and anterodistal corner, width 1.7 times as long as depth; basis thick and short, anterior margin bare, submargin with setae, posterior margin with 7 long setae; posterodistal corner of carpus with serrate robust seta; propodus stout, subtriangular, palmar margin with 14 robust setae in 2 rows, some distally notched; posterior margin of dactylus dentate (Fig. 4D). Gnathopod 2 (Fig. 4E, F) with rounded subquadrate coxal plate, with setae on its anterior to ventral margins, width 1.3 times as long as depth; basis slender with short setae on anterior margin, posterior margin with 8 long setae; posterodistal corner of carpus with serrate robust seta; propodus more slender than that of gnathopod 1, palmar margin with 14 robust setae in 2 rows, some distally notched; posterior margin of dactylus dentate (Fig. 4G).

**Figure 5.**
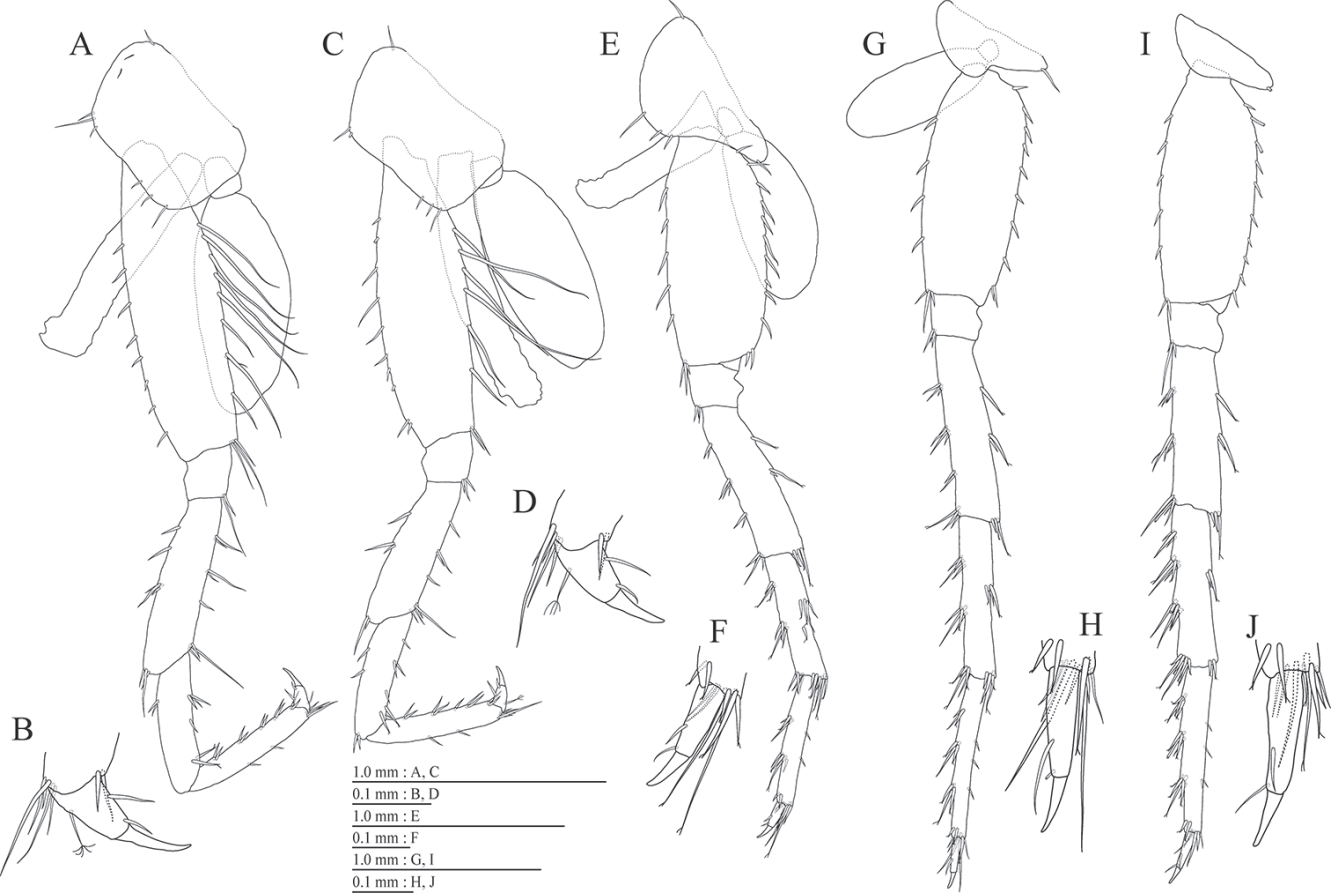
*Pseudocrangonyx wonkimi* sp. nov., holotype, female (BL = 8.9 mm). **A** Pereopod 3, lateral view **B** dactylus of pereopod 3, lateral view **C** pereopod 4, lateral view **D** dactylus of pereopod 4, lateral view **E** pereopod 5, lateral view **F** dactylus of pereopod 5, lateral view **G** pereopod 6, lateral view **H** dactylus of pereopod 6, lateral view **I** pereopod 7, lateral view **J** dactylus of pereopod 7, lateral view.

Pereopod 3 (Fig. 5A) with subquadrate coxal plate bearing setae on anterior to ventral margins, width 1.5 times as long as depth; anterior and posterior margins of basis with short and long setae, respectively; merus, carpus, and propodus in length ratio of 1.0 : 0.7 : 0.7; posterior margin of dactylus with 2 setae (Fig. 5B). Pereopod 4 (Fig. 5C) with subquadrate coxal plate bearing setae on anterior to ventral margins, width 1.7 times as long as depth; anterior and posterior margins of basis with setae; merus, carpus, and propodus in length ratio of 1.0 : 0.8 : 0.8; posterior margin of dactylus with 2 setae (Fig. 5D). Pereopod 5 (Fig. 5E) with weakly bilobed coxal plate bearing setae on anterior to posterior lobes; anterior and posterior margins of basis with setae; merus, carpus, and propodus in length ratio of 1.0 : 0.8 : 0.8; anterior margin of dactylus with 2 setae (Fig. 5F). Pereopod 6 (Fig. 5G) with weakly bilobed coxal plate bearing setae on posterior lobes; anterior and posterior margins of basis with setae; merus, carpus, and propodus in length ratio of 1.0 : 0.9 : 0.9; anterior margin of dactylus with 2 setae (Fig. 5H). Pereopod 7 (Fig. 5I) with posteriorly tapering coxal plate, ventral margin weakly concave, with seta on posterodistal corner; anterior and posterior margins of basis with short setae; merus, carpus, and propodus in length ratio of 1.0 : 1.0 : 1.0; anterior margin of dactylus with 2 setae (Fig. 5J).

Coxal gills (Fig. 4E, 5A, C, E, G) on gnathopod 2 and pereopods 3–6; sternal gills absent.

Brood plates (Fig. 4E, 5A, C, E) slender with numerous setae, on gnathopod 2 and pereopods 3–5.

**Figure 6.**
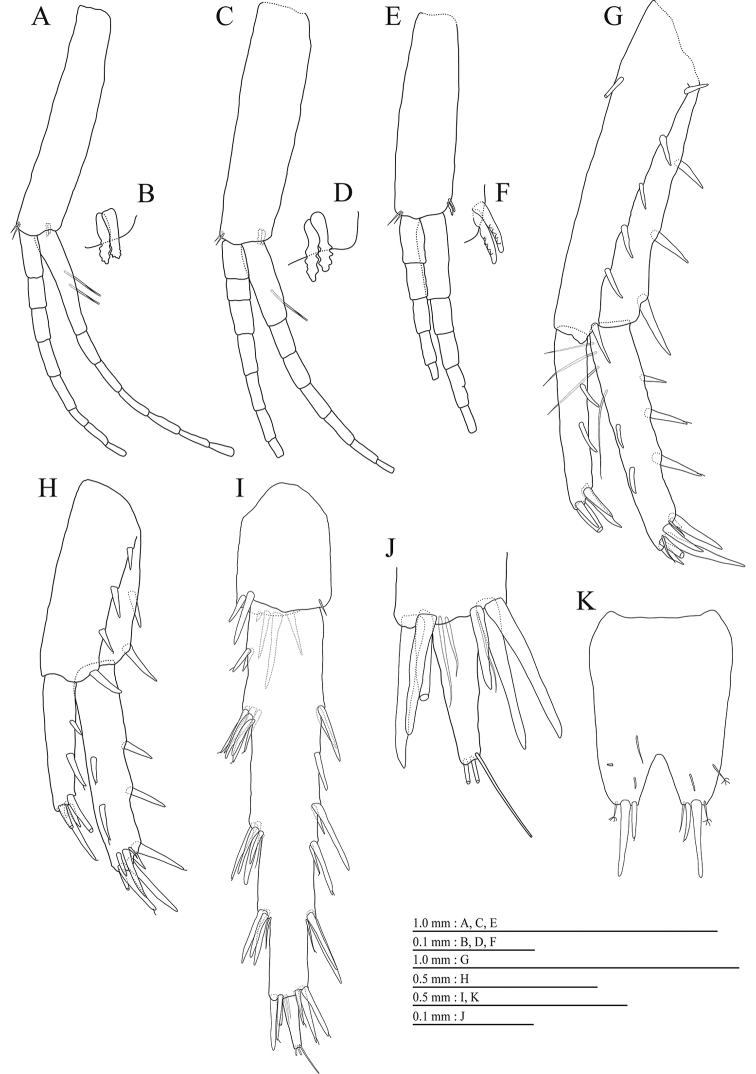
*Pseudocrangonyx wonkimi* sp. nov., holotype, female (BL = 8.9 mm). **A, C, E** Pleopods 1–3, lateral view, plumose setae on rami omitted **B, D, F** retinacula on peduncle of pleopod 1–3, lateral view **G** uropod 1, dorsal view **H** uropod 2, dorsal view **I** uropod 3, dorsal view **J** terminal article of uropod 3, dorsal view **K** telson, dorsal view.

Peduncles of pleopods 1–3 (Fig. 6A, C, E) lacking marginal setae, anterodistal corners with 2 setae. Pleopods 1–3 with paired retinacula (Fig. 6B, D, F). Pleopods 1 and 2 with bifid seta (clothes-pin seta) on inner basal margin of inner ramus; pleopods 1–3 inner ramus 7-, 7-, and 5-articulate, respectively; pleopods 1–3 outer ramus 8-, 7-, and 5-articulate, respectively.

Uropod 1 (Fig. 6G) with basofacial seta on peduncle; inner ramus 0.7 times as long as peduncle, inner and outer margins with 3 and 2 robust setae, respectively, basal part with 4 slender setae; outer ramus 0.8 times as long as inner ramus, with 2 outer marginal robust setae. Uropod 2 (Fig. 6H) with inner ramus 1.1 times as long as peduncle, inner and outer margins each with 2 robust setae; outer ramus 0.7 times as long as inner ramus, with 2 outer marginal robust setae, respectively. Uropod 3 (Fig. 6I, J) with peduncle 0.3 times as long as outer ramus; inner ramus absent; outer ramus 2-articulate, proximal article with robust setae, terminal article 0.1 times as long as proximal article, with 3 distal setae.

Telson (Fig. 6K) length 1.57 times as long as wide, cleft for 25.0% of its length, each telson lobe apical with penicillate seta and 2 robust setae.

**Figure 7.**
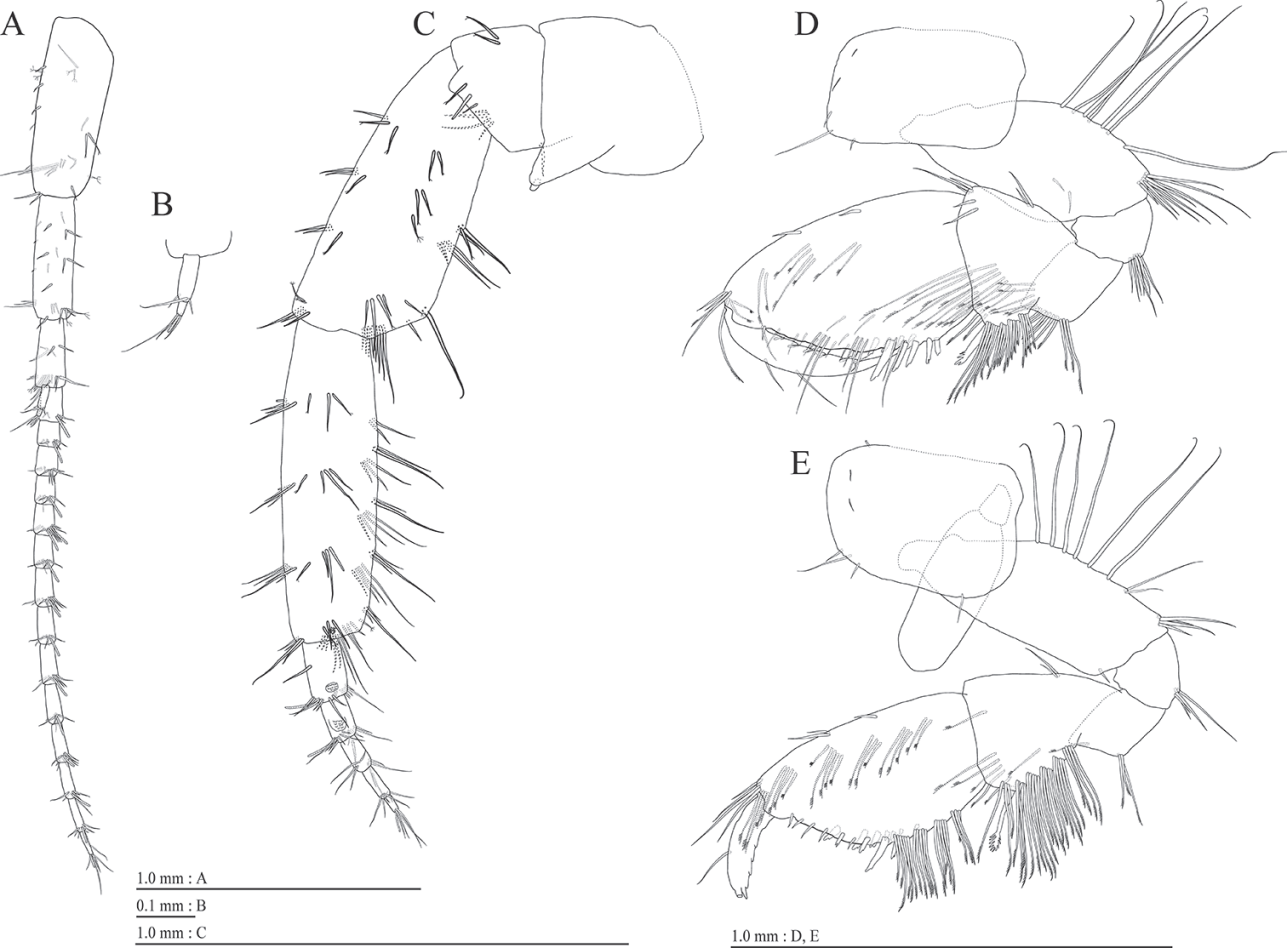
*Pseudocrangonyx wonkimi* sp. nov., paratype, male (BL = 8.3 mm). **A** Antenna 1, medial view **B** accessory flagellum of antenna 1, medial view **C** antenna 2, medial view **D** gnathopod 1, lateral view **E** gnathopod 2, lateral view.

**Male** (NNIBRIV36158, BL = 8.3 mm). Antenna 1 (Fig. 7A, B) 0.36 times as long as body length, primary flagellum 14-articulate, 1 aesthetasc on some articles. Antenna 2 (Fig. 7C) 0.66 times as long as antenna 1; flagellum 0.33 times as long as peduncular articles 4 and 5 combined, consisting of 5 articles, articles 1 and 2 with calceolus.

Gnathopod 1 (Fig. 7D) carpus with serrate seta on posterodistal corner; palmar margin of propodus with 10 robust setae in 2 rows, some distally notched. Gnathopod 2 (Fig. 7E) carpus with serrate seta on posterodistal corner; palmar margin of propodus with 11 robust setae in 2 rows, some distally notched.

**Figure 8.**
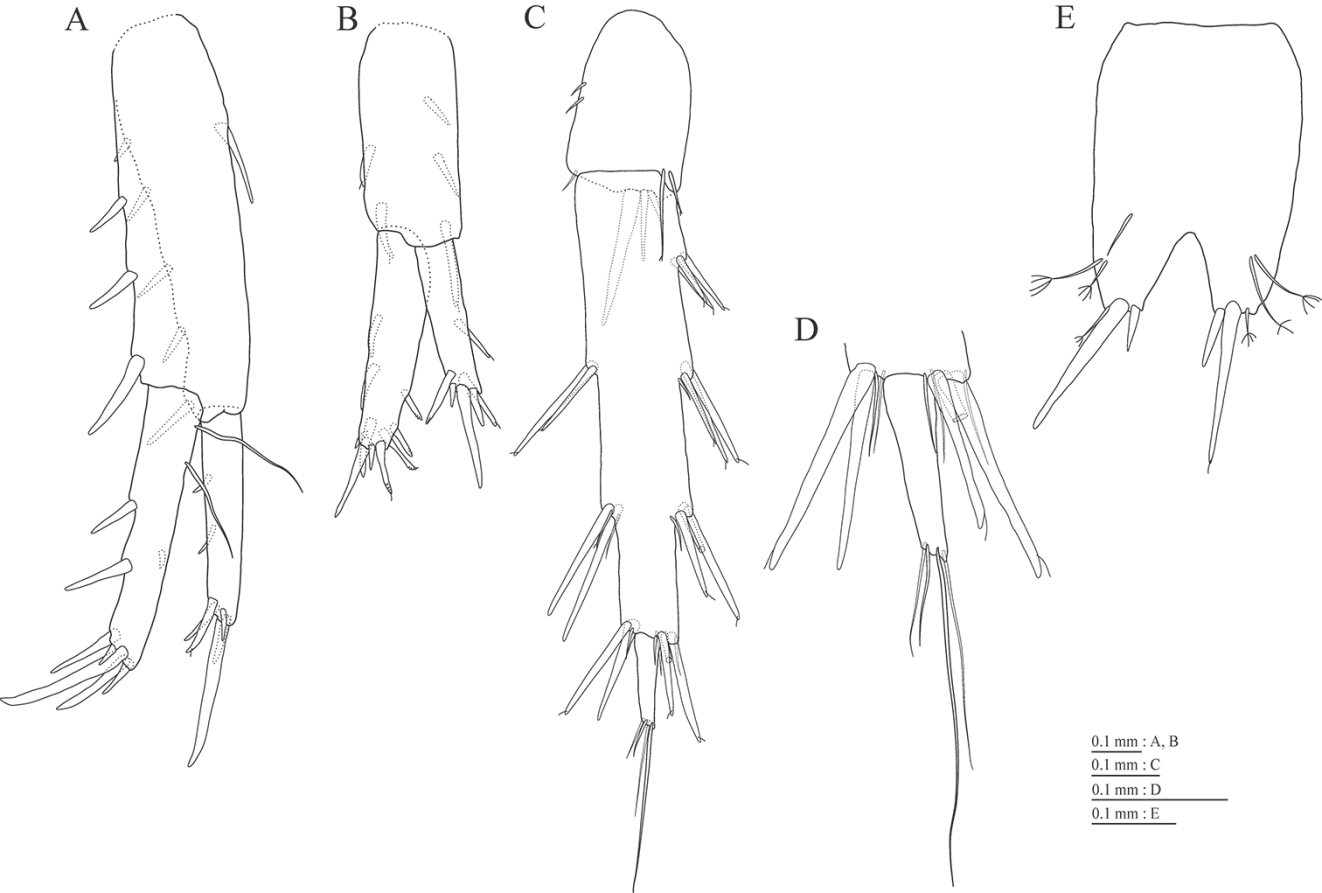
*Pseudocrangonyx wonkimi* sp. nov., paratype, male (BL = 8.3 mm). **A** Uropod 1, ventral view **B** uropod 2, ventral view **C** uropod 3, ventral view **D** terminal article of uropod 3, ventral view **E** telson, dorsal view.

Uropod 1 (Fig. 8A) with inner ramus 0.7 times as long as peduncle; inner and outer margins with 2 and 1 robust setae, respectively, basal part with 2 slender setae; outer ramus with 2 marginal robust setae. Uropod 2 (Fig. 8B) with peduncle 0.95 times as long as inner ramus; inner ramus 1.4 times as long as outer ramus, distal part with 3 serrate, 4 simple robust setae. Uropod 3 (Fig. 8C, D) with outer ramus terminal article 0.2 times as long as proximal article.

Telson (Fig. 8E) length 1.38 times as long as wide, cleft for 27.4% of its length.

#### Distribution.

Known only from the type locality.

#### Etymology.

The name of the new species is dedicated to Prof. Won Kim (Seoul National University, South Korea), who has significantly contributed to our knowledge of crustaceans in South Korea.

#### DNA sequences.

Sequences of COI gene (MT316534 and MT316535) were determined from two specimens (NNIBRIV35119 and NNIBRIV36158).

#### Molecular analyses.

**Figure 9.**
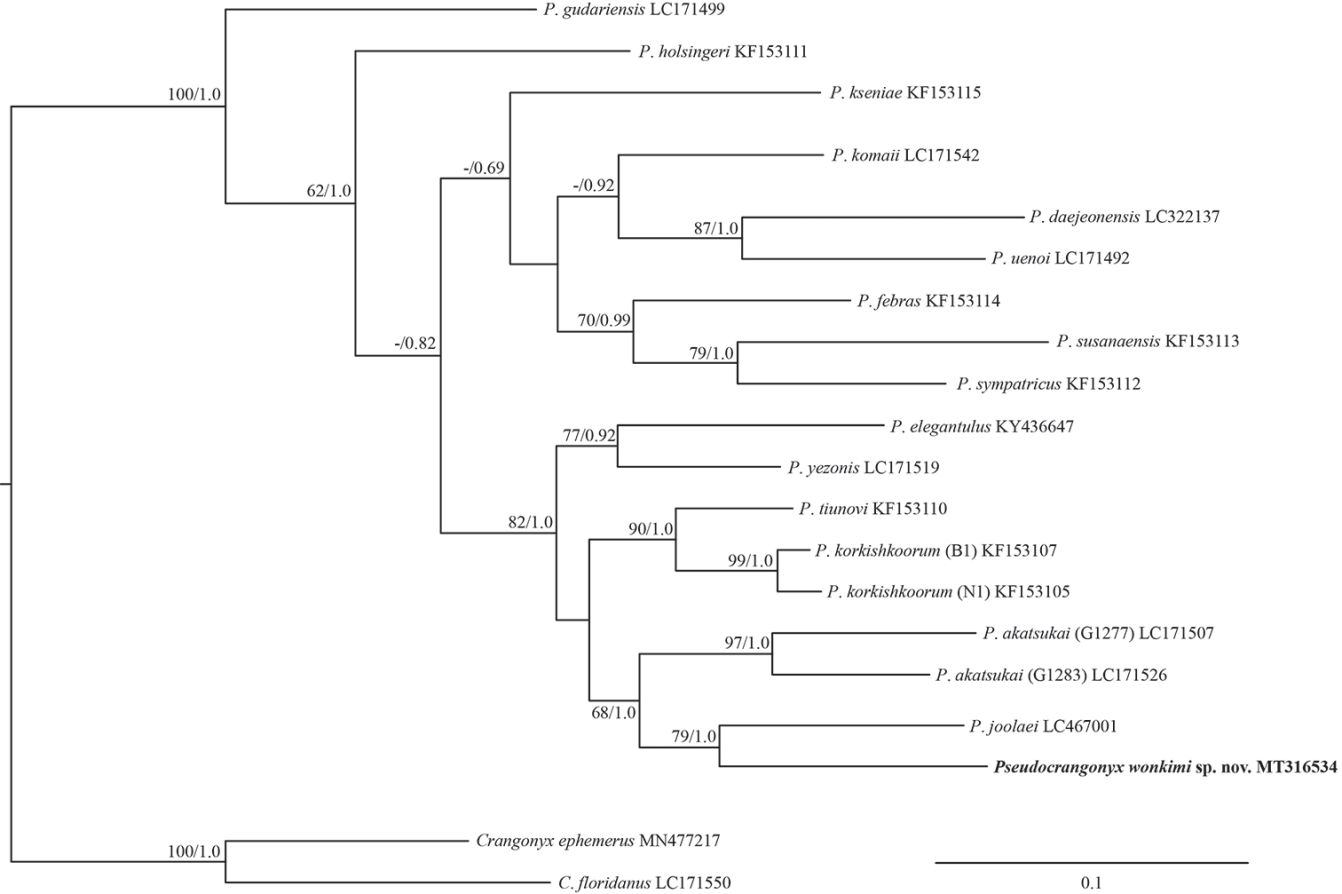
Maximum likelihood and Bayesian inference analyses based on mitochondrial COI sequences. Numbers on nodes represent bootstrap values for maximum likelihood and Bayesian posterior probabilities.

The topologies of the BI and ML trees were identical (Fig. 9). *Pseudocrangonyx wonkimi* sp. nov. and *P. joolaei*Lee et al., 2020 showed the closest relationship.

#### Remarks.

We revealed that *Pseudocrangonyx wonkimi* sp. nov. is most closely related to *P. joolaei*Lee et al., 2020 based on molecular analyses. The genetic distance between these two species was 15.1% for the COI gene, and this distance is larger than that between members of two distinct species among the other congeners examined. *Pseudocrangonyx wonkimi* sp. nov. is distinguished from *P. joolaei* in having the following features (features of *P. joolaei* in parentheses): 1) sternal gill absent (present), 2) maxilla 1 inner plate with 4 (6) plumose setae, 3) carpus of gnathopods 1 and 2 each with serrate robust seta (with 2–3) on the posterodistal corner, 4) inner basal margin of inner rami of pleopod 3 without (with) bifid seta, and 5) less than 10 (more than 10) articles on the rami of pleopods.

*Pseudocrangonyx wonkimi* sp. nov. is morphologically similar to *P. akatsukai* Tomikawa & Nakano, 2018 in having 1) eyes completely absent, 2) sternal gill absent 3) urosomite 1 with ventral robust seta, 4) antenna 2 with calceoli in both sexes 5) carpi of gnathopods 1 and 2 with serrate robust setae on posterodistal corner, and 6) inner rami of pleopods with bifid setae on inner margin. However, the former is distinguished from the latter by the following features (features of *P. akatsukai* in parentheses): 1) pereonites 3–5 (1–7) with short dorsal setae, 2) antenna 1 shorter (longer) than as long as body length half, 3) male antenna 2 flagellum 0.33 (0.53) times as long as peduncular articles 4 and 5 combined, 4) carpi of gnathopods 1 and 2 with 1 (with 3–5) serrate robust setae on posterodistal corner, 5) length ratio of merus, carpus, propodus 1.0 : 1.0 : 1.0 (1.0 : 1.1 : 1.1) of pereopod 7, and 6) telson cleft for 25.0–27.4% (6.6–12.3%) of length.

*Pseudocrangonyx wonkimi* sp. nov. is similar to *P. elegantulus* Zhao & Hou, 2017 in having 1) urosomite 3 dorsal margin without seta, 2) sternal gill absent, 3) accessory flagellum of antenna 1 subequal first article of primary flagellum, and 4) antenna 2 with calceoli in both sexes. However, *P. wonkimi* is distinguished from the latter by the following features (features of *P. elegantulus* in parentheses), 1) pereonites 3–5 with (1–6 without) dorsal margin setae, 2) basal part of inner ramus of female uropod 1 with 4 (1) slender setae, 3) uropod 1 peduncle inner marginal with 3 (1) robust setae, 4) terminal article of uropod 3 almost reaching (fully exceed) robust setae on the distal part of the proximal article, and 5) carpus of male gnathopod 2 with (without) serrate robust seta on posterodistal corner. *Pseudocrangonyx wonkimi* sp. nov. is similar to *P. shikokunis* Akatsuka & Komai, 1922 in having 1) eyes absent, 2) mandible palp article 3 longer than article 2, and 3) carpi of gnathopods 1and 2 with serrate setae on the posterodistal corners. The new species is distinguished from the latter by the following features (features of *P. shikokunis* in parentheses), 1) antenna 1 shorter (longer) than half of the body length, 2) maxilla 1 inner plate with 4 or fewer setae (with 5 setae), and 3) male telson cleft for 27.4% (11.7%) of length. The new species is similar also to *P. cavernarius* Hou & Li, 2003 in having 1) body size about 8.0 mm, and 2) maxilla 1 inner plate with 4 plumose setae. It differs from *P. cavernarius* Hou & Li, 2003 by the following features (features of *P. cavernarius* in parentheses), 1) antenna 2 calceoli present (absent), 2) mandible palp article 3 longer (shorter) than article 2, 3) urosomite 3 dorsal margin without (with) setae, and 4) telson each lobe with (without) setae.

## Supplementary Material

XML Treatment for Pseudocrangonyx wonkimi
